# Iron Chelation Therapy with Deferasirox in the Management of Iron Overload in Primary Myelofibrosis

**DOI:** 10.4084/MJHID.2014.042

**Published:** 2014-06-01

**Authors:** Elena Maria Elli, Angelo Belotti, Andrea Aroldi, Matteo Parma, Pietro Pioltelli, Enrico Maria Pogliani

**Affiliations:** Hematology Division, San Gerardo Hospital, Monza, Italy

## Abstract

Deferasirox (DSX) is the principal option currently available for iron-chelation-therapy (ICT), principally in the management of myelodysplastic syndromes (MDS), while in primary myelofibrosis (PMF) the expertise is limited. We analyzed our experience in 10 PMF with transfusion-dependent anemia, treated with DSX from September 2010 to December 2013. The median dose tolerated of DSX was 750 mg/day (10 mg/kg/day), with 3 transient interruption of treatment for drug-related adverse events (AEs) and 3 definitive discontinuation for grade 3/4 AEs. According to IWG 2006 criteria, erythroid responses with DSX were observed in 4/10 patients (40%), 2 of them (20%) obtaining transfusion independence. Absolute changes in median serum ferritin levels (Delta ferritin) were greater in hematologic responder (HR) compared with non-responder (NR) patients, already at 6 months of ICT respect to baseline. Our preliminary data open new insights regarding the benefit of ICT not only in MDS, but also in PMF with the possibility to obtain an erythroid response, overall in 40 % of patients. HR patients receiving DSX seem to have a better survival and a lower incidence of leukemic transformation (PMF-BP). Delta ferritin evaluation at 6 months could represent a significant predictor for a different survival and PMF-BP. However, the tolerability of the drug seems to be lower compared to MDS, both in terms of lower median tolerated dose and for higher frequency of discontinuation for AEs. The biological mechanism of action of DSX in chronic myeloproliferative setting through an independent NF-κB inhibition could be involved, but further investigations are required.

## Introduction

Primary myelofibrosis (PMF) is a clonal chronic myeloproliferative neoplasm characterized by reactive bone marrow fibrosis, osteosclerosis, angiogenesis and an abnormal cytokine expression leading to extramedullary hematopoiesis (EMH) and progressive cytopenia. Clinical manifestations of PMF include constitutional symptoms, marked hepatosplenomegaly secondary to EMH, uncontrolled myeloproliferation manifesting with marked leukocytosis or thrombocytosis or progressive cytopenias.^1^ Current standard therapies and also experimental approach with JAK2 inhibitors (as Ruxolitinib) focus on symptom management, spleen enlargement reduction and optimizing cell counts, and a mainstay of therapy is transfusion support.^2^ Patients with PMF frequently may develop anemia from decreased marrow reserve, ineffective erythropoiesis, splenic sequestration and myelosuppressive medications.^3^ Many patients eventually require red blood cell (RBC) transfusions, which may lead to iron overload (IOL) from transfused blood and increased iron absorption;^3^ significant IOL may occur after as few as 20 RBC units,^4^ and transfusion dependent (TD) patients may develop cardiac, hepatic and endocrine dysfunction.^5^,^6^ Organ damage secondary to TD has been described as detrimental for survival in PMF, and it presents a prognostic relevance independently from the International Prognostics Scoring System, IPSS (which considers age over 65 years, leukocytosis higher than 25 × 10^9^/l, Hemoglobin level lower than 10 g/dl, peripheral blasts equal to or higher than 1% and presence of constitutional symptoms), or other prognostic score.^7^ At last, IOL represents a risk factor for early hepatotoxicity, and it impacts on survival in patients with PMF undergoing allogeneic hematopoietic cell transplantation.^8^ Iron related organ toxicity is mediated in part by deposition of iron into tissues and organs, and in part by the chronic exposure to non-transferrin bound iron (NTBI), which leads to the formation of labile plasma iron (LPI) and reactive oxygen species (ROS) capable of damaging lipids, proteins and nucleic acids, thereby possibly provoking apoptosis^9^,^10^ and mutagenesis.^11^ In particular, LPI represents the most toxic fraction of NTBI, which is both redox active and chelatable and, over time, sustained levels of LPI may compromise organ function and overall survival. LPI is taken up into cells leading to an increased labile iron pool with rapid generation of ROS. The iron pool has, therefore, been regarded as one of the main regulators of the production of ROS in cells. Oxidative stress leads to oxidation of proteins, lipids and DNA, as well as suppression of the self-renewal of the hematopoietic stem cells, a decrease in the number of these cells and increased apoptosis and organ damage.^12^

Transfusion-induced IOL is a frequent problem that clinicians have to face in the management of patients affected by hematologic neoplasms.^13^–^15^ For example in myelodysplastic syndromes (MDS), many recent studies demonstrated that TD patients in comparison to transfusion independent (TI) ones have a lower survival which is proportional to the degree of transfusion dependence.^16^–^17^

To limit the toxicity of excess iron, patients may receive iron chelation therapy (ICT). The benefits of ICT in patients with thalassemia major and IOL are well established^18,^ and ICT has been shown to reduce LPI levels and oxidative stress^14^ in TD patients. More recent studies suggest that administration of ICT may improve survival in patients with MDS and IOL.^19^,^20^

Deferasirox (DSX) is the principal option currently available for oral ICT.^21^ DSX has been demonstrated to decrease NTBI, to maintain or reduce body iron (as assessed by serum ferritin) and to have a good tolerability profile with no severe adverse effects in pre-treated or therapy-naïve MDS patients.^21^,^22^ This oral ICT also seems to induce a hematologic improvement that leads to a significant reduction or complete interruption of blood transfusions in MDS patients, in addition to improving the survival.^23^ Hematologic responses also in term of increase in platelet and neutrophil count have been observed in MDS setting.^24^ The exact mechanism of the hematologic response to ICT is unknown, and the relationship between DSX and erythroid improvement has yet to be elucidated. It has been hypothesised that DSX acts not only reducing the high levels of LPI into the plasma but also in the bone marrow through a direct and protracted effect on the microenvironment and against the neoplastic clone. As compared to other iron chelators, DSX is a potent NF-kB inhibitor and is able to increase glutathione (GSH) in red blood cells, thus protecting them from oxidative insults.^25^ Despite these observations, the role of ICT in PMF remains largely undefined, and a few reports are present in the literature regarding this specific setting of Philadelphia-negative chronic myeloproliferative neoplasm (MPN Ph-),^26^–^29^ reflecting the limited expertise in this field. Few data have shown a survival benefit associated with the use of ICT in patients with TD anemia and PMF,^29^ similar to MDS, but more importantly there are no sufficient studies that have examined the effectiveness in terms of hematological response, particularly erythroid response, together with the safety profile in patients with PMF. Here we reported our experience in TD-PMF patients treated with DSX from September 2010 to December 2013, in order to evaluate the efficacy and safety profile of this approach in MPN Ph- setting.

## Material and Methods

We identified in our MPN Ph- database, 154 patients affected by myelofibrosis, referred to our division from 1990 to 2012. We identified 47 patients with PMF (30,5%) presenting TD anemia at onset or during follow-up of disease; of whom, we analyzed 10 TD-PMF patients treated with oral DSX, from September 2010 to December 2013, starting from a dose of 10 mg/kg/day, up to the maximum tolerated dose. In this way, all the patients were evaluable for toxicity and hematologic response (≥ 6 months of treatment). Criteria for initiating ICT were an estimated life expectancy of at least 1 year and at least one of elevated ferritin level (over 1000 μ/l), transfusion of at least 20 RBC units, or organ dysfunction from IOL, refractoriness and/or absence of concomitant therapy with stimulant erythropoietic agents (recombinant erythropoietin, steroids, immunosuppressive therapy). Clinical evidence of IOL was determined retrospectively as organ dysfunction in the absence of other etiology. In particular cardiac dysfunction was defined as left ventricular enlargement or decreased ejection fraction, clinical signs of systolic or diastolic dysfunction or arrhythmia. Hepatic dysfunction included clinical signs of liver disease or alanine aminotransferase (ALT) or aspartate aminotransferase (AST) greater than 1.5 times the upper limit of normal. Endocrin dysfunction included glucose intolerance or diabetes, and thyroid stimulating hormone level above the upper limit of normal. 4/10 patients at baseline were assessed by noninvasive liver iron concentration (LIC) measurement using R2-magnetic resonance imaging for evaluation of hepatic damage; only one patient with severe serological hepatic dysfunction underwent to hepatic biopsy with histological confirmation of iron damage. Unfortunately, none of these patients was monitored over time using R2-magnetic resonance imaging, in order to re-evaluate the hepatic iron deposits after ICT.

### Assessment and statistical methods

We evaluated the efficacy of ICT in PMF patients in term of reduction in serum ferritin levels and hematologic responses. Efficacy of ICT was assessed evaluating the changes from baseline in serum ferritin levels after 6, 12 and 18 months of treatment with DSX and at the end of treatment. Details of ongoing RBC transfusion were recorded throughout the study. Transfusional iron intake, expressed in mg of iron, was calculated as the total amount of pure RBC transfused × 1.08.^30^

The IWG 2006 criteria^31^ were used to evaluate erythroid, platelet and neutrophil response during DSX treatment. Time to hematologic response was assessed as the number of days from the first dose of DSX to the onset of hematologic response. According to erythroid response, defined as complete response (CR: transfusion independent patients), partial response (PR: reduction in transfusion requirement or increases in Hb levels) or absence of response, the patients were divided into 2 subgroups: hematologic responder (HR: CR + PR) and non-responder (NR) patients.

Non parametric analysis, Fisher’s exact test and Mann-Whitney test, were performed to evaluate, respectively, the qualitative and quantitative variables in HR and NR patients of collection. In order to evaluate the efficacy of ICT, the non-parametric Wilcoxon signed-rank test was used to calculate *P*-values for changes in serum ferritin levels, in the course of ICT compared to baseline, in each HR e NR group; the Mann-Whitney test was used to compare the difference in the changes of serum ferritin levels of the two groups between them.

We considered the safety and tolerability of treatment, in term of transient or definitive discontinuation of DSX for drug-related adverse events (AEs) and median tolerated dose of DSX. Safety and tolerability were evaluated throughout the study by monitoring the incidence and type of adverse events (AEs) and by assessing routine laboratory parameters. AEs were assessed according to CTCAE Version 4 (2009) definition. Overall survival (OS) was defined as the time from the date of PMF diagnosis to the date of death from any cause. AML transformation (PMF-BP) was defined as the appearance of > 10% circulating blasts in the peripheral blood and/or at least 20% blasts in the bone marrow.^26^ Patients still alive were censored at the last known date of follow up.

This study was notified and performed with the requirements of the San Gerardo Hospital Institutional Research Ethics Board. All procedures were followed according to the Helsinki Declaration.

## Results

We treated from September 2010 to December 2013, 10 TD-PMF patients, with a median age of 70.5 (range 55–81) years at onset of ICT. Principal clinical and laboratory features are summarized in Table 1. Median hematologic at diagnosis were: Hemoglobin 8.95 (7.3–9.6) g/dl, platelet count 228 (12–1050) × 10^9^/l and WBC count 8.96 (2.71–23.8) × 10^9^/l. The median baseline serum ferritin levels was 1702 (range 1173–3198) μ/l.

As showed in Table 2, starting dose of DSX was 10 mg/kg/day, increasing up to the maximum tolerated dose, for a median time of exposure to ICT of 11 (range 1–33) months. The median dose tolerated of the DSX was 750 (range 500–1500) mg/day, i.e 10 mg/kg/day. Treatment with DSX started after a median interval from diagnosis of 43,5 (range 7–207) months.

Before starting ICT, the median number of RBC transfusions received by patients was 28 (range 10–150) RBC units/patient. The corresponding median transfusional iron intake was 0.27 (range 0.07–1.11) mg/kg/day. No patients presented cardiac or endocrine dysfunction at baseline. Three patients (30%) showed hepatic dysfunction at serological test or iron hepatic damage at R2-magnetic resonance imaging or hepatic biopsy at onset of ICT.

We reported only 3 transient interruption of treatment for grade 2 extra-hematological toxicity: in particular 1 cutaneous rash, 1 diarrhea and 1 transaminitis. 5/10 patients (50%) experienced a definitive discontinuation of the drug for grade 3/4 AEs (1 hepatitis, 1 cutaneous rash with mucous membrane ulceration, 1 intestinal malabsorption and 2 renal failure). Drug-related AEs were reported after a median time of 135 (range 15–612) days from start of ICT. Two patients interrupted DSX for PMF-BP, 1 patient developed PMF-BP after discontinuation of DSX. Overall, only 3/10 patients (30%) continued permanently oral ICT.

### Effect of DSX on hematologic parameters

#### HR and NR patients

**Figure 1:**
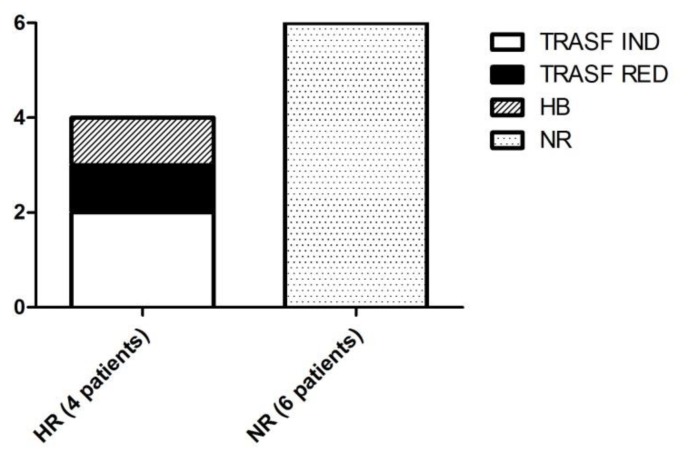
Erythroid response in hematologic responder (HR) patients (n=4) treated with DSX, comprising Hb improvement (Hb response: n=1), reduction of transfusion requirements (TRASF RED: n=1) or transfusion independence (TRASF IND: n=2); NR: non responder patients (n=6).

According to IWG 2006 criteria, erythroid responses with DSX were observed in 4/10 patients (40%), after a median of 150 (range 94–352) days; in particular we reported 2 PR: 1 patient with reduction in transfusion requirements and 1 patient with hemoglobin improvement. Two patients (20%) obtained transfusion independence, ie CR, as showed in Figure 1. Six patients did not achieve any hematologic response. One patient achieved an improvement of liver function. At last, we did not record platelet and neutrophil response during DSX treatment. The demographics and principal characteristics in HR and NR patients were summarized in Table 1.

The HR patients were younger at onset of ICT (61.5 vs 74.5 years, p = 0.007), the median time from diagnosis to start of ICT was longer in HR group (69.5 vs 16.5 months, p = 0.05) even if the distribution of IPSS score was similar in two groups; transfusion history prior to ICT, both in terms of absolute number of RBC units/patient and transfusional iron intake was not significantly different in HR respect to NR patients (40.5 vs 28 RBC units/patient and 0.21 vs 0.29 mg/kg/day, respectively). The median daily dose of DSX received by patients was similar in each group (HR: 12 mg/kg/day vs NR: 10 mg/kg/day, p = NS), even if we noted a trend for a shorter median exposure time at DSX in NR patients compared to HR patients (6.5 vs 14.5 months). Of note, there was no difference in drug exposure between the two groups in the first 6 months of ICT: only 1 patient in HR group (for cutaneous rash) and 2 NR patients discontinued DSX within 6 months of treatment (1 patient for hepatitis and 1 patient for PMF-BP). Furthermore, transfusional iron intake during ICT was also similar in two groups (0.65 vs 0.47 mg/kg/day, respectively in HR and NR group).

**Figure 2:**
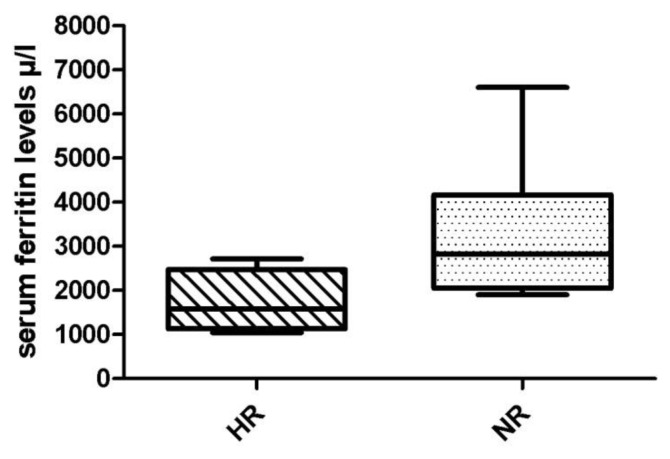
Serum ferritin levels in hematologic responder (HR) and non-responder (NR) patients at the end of iron chelation therapy (ICT).

The median serum ferritin levels at baseline were comparable in both HR and NR patients (1988 vs 1702 μ/l, respectively). Analyzing serum ferritin levels at 6, 12 and 18 months from start of ICT, median serum ferritin levels at 6 months, respect to baseline, were already reduced in HR patients (ferritin level at baseline 1988 μ/l, and ferritin level at 6 months 1756 μ/l) without reaching statistical significance. In NR patients, it was not observed any reduction in the serum ferritin levels at baseline respect of any time of ICT. In fact, this group presented a significant increase of serum ferritin levels at any time until the end of treatment (p = 0.03), comparing with HR patients, as reported in Figure 2.

**Figure 3:**
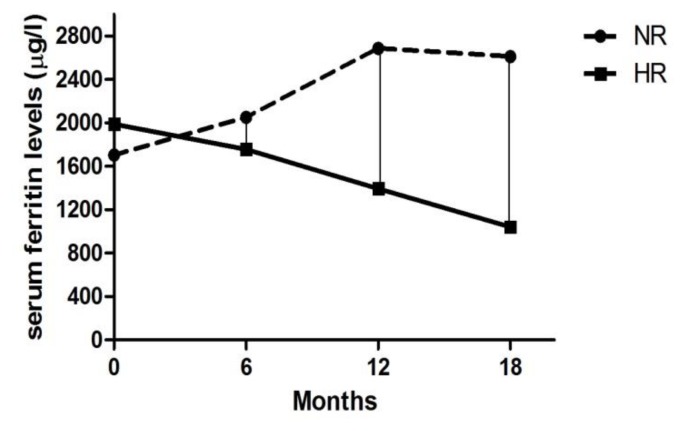
Absolute change in median serum ferritin levels from baseline in hematologic responder (HR) and non-responder (NR) patients at 6, 12, 18 months from start of iron chelation therapy (ICT).

**Figure 4:**
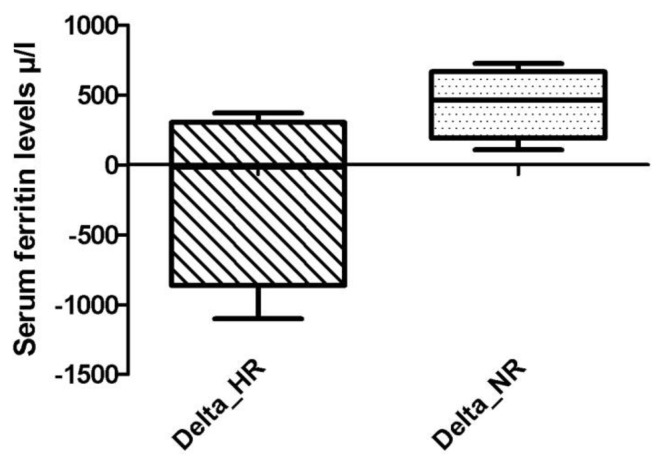
Median change from baseline in serum ferritin levels (Delta ferritin) at 6 months in hematologic responder (HR) and non-responder (NR) patients.

In other words, the reductions in median serum ferritin levels were obtained at any time during ICT only in HR patients, as showed in Figure 3. Furthermore, comparing two groups, the difference in the median changes of serum ferritin levels (Delta ferritin evaluation) was statistically significant already at 6 months of ICT, as showed in Figure 4: HR patients experienced at 6 months of ICT a significant reduction in serum ferritin levels of – 17.5 μ/l (range: - 1102 to 370 μ/l) compared with an increase of + 464 μ/l (range: 108 to 723 μ/l) in NR group (p = 0.028).

In patients who met the criteria for hematologic erythroid response, the frequency of drug-related AEs was similar to NR patients, but the incidence of definitive discontinuation of ICT for AEs was higher in NR group (75% vs 25%). However, the median time of onset of AEs was later in NR patients compared to HR group (248 vs 119 days).

PMF-BP evolution was reported only in 3 NR patients. Finally, HR patients seem to present a better OS than NR patients (median OS: 93 vs 35.5 months, respectively). OS at 5 years was greater in HR respect to NR patients (75% vs 33%, respectively). There were 7 deaths, 5 of them in NR patients. Causes of the 5 deaths in NR were: 3 PMF-BP, 1 sepsis and 1 bleeding. The 2 deaths in HR patients were 1 bleeding and 1 sepsis occurred 45 days after bone marrow transplantation.

## Discussion

PMF is a myeloproliferative neoplasm frequently complicated by TD anemia. Given the detrimental effects of anemia and of IOL due to a prolonged transfusional support, any treatment able to improve anemia and transfusion dependence could have a significant impact on patient’s quality of life and life expectancy.^26^ Our preliminary data open new insights regarding the benefit of ICT not only in MDS, but also in PMF patients with TD anemia, with the possibility to obtain a partial or complete erythroid response, overall in 40 % of them.

Several emerging lines of evidence actually indicate that ICT can improve hematopoiesis and leads to a reduction or abolition of transfusion dependence in PMF.^26^–^29^,^32^ Therefore these data are very sparse and mainly deriving from single case descriptions, but they are suggestive of a real biological phenomenon. A similar positive impact on transfusion dependence has been also described in patients with MDS thus suggesting the absence of a specific correlation between hematopoietic improvement due to ICT and the type of disease.^19^,^23^,^24^ Our study represents the first attempt to assess the efficacy, safety and potential benefit of ICT in this specific setting of patients, although with the limitation of a small series. However, we stress as in the inclusion criteria, we deliberately excluded the patients who were concomitantly in treatment with stimulant erithropoietic agents, in order to remove any confounding factor on the hematologic response, with consequent reduction of analyzed cases.

Our results in PMF patients, in term of improvement of hematopoiesis, reproduce those obtained in MDS setting on a larger series of patients. Gatterman et al.^24^ reported a post-hoc analysis of haematological response to DSX in a cohort of 247 iron-overloaded patients with MDS enrolled in the EPIC trial. Erythroid, platelet and neutrophil responses were observed in 21.5% (53/247), 13.0% (13/100) and 22.0% (11/50) of the patients after a median of 109, 169 and 226 days, respectively. Of the patients with an erythroid response, 28 (11.3%) had only a transfusion response and 22 (8.9%) had only a haemoglobin response. Three patients (1.2%) had both transfusion and haemoglobin responses.

In our study, erythroid responses were observed in a high proportion of patients (40%), after a median time of 150 days after starting DSX. Of the patients with an erythroid response, 2 patients obtained a transfusion independence (20%), 1 patient (10%) had a partial transfusion response and 1 patient (10%) had only a improvement in haemoglobin levels. Conversely, we did not record platelet and neutrophil response during DSX treatment.

ICT therefore may have a role in the management of anemia in PMF, in all stages of the disease, in patients treated with conventional cytoreductive therapy but especially in consideration of new drugs that are now used in this setting, ie JAK2 inhibitors. One of the main side effects of these drugs, linked to the intrinsic mechanism of action in JAK2-STAT pathway, seems to be the induction or the worsening of the degree of anemia in PMF patients, especially in the first 6 months of therapy; consequently the use of RBC transfusion is critical in order to avoid the tapering of drug. ICT in these patients can reduce the amount of IOL and prevent organ damage, keeping the effective dose of the drug stable and potentially contributing to the potential hematologic improvement with JAK2 inhibitors.

The tolerability of the drug seems to be lower in PMF compared to MDS patients, both in terms of lower median tolerated dose (10 mg/kg/day) and of a higher frequency of discontinuance for drug related AEs (50%). Nevertheless, among the patients treated with DSX, we identified a subgroup that responds to ICT, achieving a hematologic improvement or even a transfusion independence. Furthermore, HR patients had a significant and progressive reduction in serum ferritin levels and we demonstrated an improvement in survival and a lower incidence of PMF-BP in this group, suggesting a potential advantage also in long term survival of treatment with DSX. In fact, based on the evaluation of serum ferritin levels in our patients in the course of ICT, hematologic responses seem to be observed in patients with greater reductions in serum ferritin levels, suggesting that hematologic response might be dependent, at least partially, on reductions in levels of body IOL. Delta ferritin evaluation at 6 months could represent a significant predictor for a different survival and PMF-BP: absolute changes in median ferritin levels was statistically greater in HR respect to NR patients and they correspond to a lower incidence of PMF-BP. PMF-BL occurred indeed only in NR patients. This phenomenon could be secondary to potentially mutagenic effect of ROS, as has been suggested in MDS.^26^,^32^ HR patients seem to have also a better survival respect to NR group (median OS 93 versus 35.5 months, respectively). This survival improvement seen in PMF patients receiving ICT is encouraging. However, because the study is retrospective and the patients’ collection is small, it is subject to the potential biases of any analysis that is non-randomized and non-controlled. To minimize the possibility of selection or referral bias favoring ICT patients, multiple baseline characteristics could be compared, showing no significant differences between groups in most factors. In our study, with the limitations mentioned above, we can say that the distribution of patients by IPSS prognostic scoring system risk and incidence of JAK2V617F mutation as well as the median serum ferritin levels at baseline, the transfusion history, the median daily iron intake before starting ICT and the median dosage of DSX were not different between two groups. We find some differences statistically significant comparing two groups in term of age: HR patients are younger at diagnosis and at the time of ICT respect to NR patients. In addition, HR patients seem to have a longer median time from diagnosis to onset of ICT (69.5 vs 16.5 months), without evidence of a more intensive transfusion requirement or daily iron intake pre-treatment. This group receives a similar daily dosage of DSX, with a trend for a longer median exposure time at the drug (14.5 versus 6,5 months) but transfusional iron intake during ICT was similar in HR and NR patients.

HR patients seem to present a lower incidence of definitive discontinuation of ICT for drug-related AEs respect to NR patients (25% versus 75%). However, the median time of onset of AEs is later NR compared to HR patients (248 vs 119 days). Therefore, the impact of drug-related AEs does not appear to affect the achievement of erythroid response, which is relatively early, as previously discussed.

All these consideration, in our opinion, could instead explain the improvement in OS in HR patients. Several possible mechanisms by which ICT can improve erythropoiesis and survival have been proposed: a direct cytoreductive effect of ICT on the neoplastic clone, a reduction of oxidative species, which are believed to correlate with inefficient erythropoiesis, or an inhibition of NF-κB leading to a reduced transcription of anti-apoptotic factors.^25^,^33^–^35^ In this specific myeloproliferative setting, the biological mechanism of action of DSX may depend mainly from the peculiar mechanism of NF-κB inhibition, independent from reactive oxygen species scavenging properties of the drug, that are common features also of other ICT, such as Deferiprone or Deferoxamine. In fact, previous studies have suggested the involvement of NF-κB pathway in the pathogenesis of the disease. In particular, Komura et al.^33^ have speculated a role of NF-κB pathway in transforming growth factor-beta1 production in PMF; Wagner-Ballon et al.^34^ have reported as Bortezomib, a proteasome inhibitor, impairs both myelofibrosis and osteosclerosis induced by high thrombopoietin levels in mice. These encouraging results *in vitro* were not confirmed by phase II clinical studies *in vivo*,^35^ probably because NF-κB pathway is not the one primarily involved in the pathogenesis of PMF. Therefore an involvement of this pathway in the mechanism of action of DSX could explain a direct action on the malignant clone during *in vivo* therapy, even if partial, inducing a hematopoietic improvement, but further investigations are required.

## Conclusions

ICT with DSX, although not routinely recommended by current guidelines of PMF management, should be proposed in clinical practice of TD patients, taking into account the possible anti-leukemic effect and the improvement of survival, besides a potential direct action of ICT in enhancing erythropoiesis of PMF patients. Further prospective and larger studies are required in order to confirm the exact role of DSX in the improvement of erythropoiesis and survival of patients with PMF and to clarify the mechanism(s) underlining this phenomenon.

## Figures and Tables

**Table 1:** Demographics and principal characteristics of all patient’s collection, hematologic responder (HR) and non-responder (NR) patients.

Characteristic	All patients (n=10)	HR patients (n=4)	NR patients (n=6)

Median age of diagnosis, years (range)	65.5 (49–81)	53 (49–61)	73 (65–81)

Male/females, n	7/3	1/3	6/0

JAK2V617f mutation, POS/NEG, n	7/3	3/1	4/2

IPSS, n:			
Low/inte-1 risk	3	1	2
Int-2/high risk	7	3	4

Median age at onset of ICT, years (range)	70.5 (55–81)	61.5 (55–65)	74.5 (68–81)

Median time from PMF diagnosis to ICT, months (range)	43.5 (7.5–207)	69.5 (40–207)	16.5 (7.5–108)

Median number of RBC transfusion in the years prior ICT (range)	28 (10–150)	40.5 (10–150)	28 (22–46)

Median daily iron intake prior ICT mg/kg/day (range)	0.27 (0.07–1.11)	0.21 (0.07–1.11)	0.29 (0.18–0.76)

Baseline hematologic parameters (range):			
Median Hb, g/dl	8.95 (7.3–9.6)	8.5 (7.3–9.4)	8.95 (7.6–9.6)
Median PLT, × 10^9^/l	228 (12–1050)	72 (12–303)	316 (16–1050)
Median WBC, × 10^9^/l	8.96 (2.71–23.8)	5.75 (2.71–10.48)	10.06 (7.54–23.8)

Median serum ferritin levels (μ/l):			
at baseline	1702	1988	1702
+ 6 months	1988	1756	2050
+ 12 months	2228	1394	2686
+ 18 months	1826	1041	2611
end of treatment	2355	1580	2822
Delta ferritin at 6 months:	+239	−17.5	+464

AEs, n	6	2	4

Median time onset AEs from ICT, days (range)	135 (15–612)	119 (79–159)	248 (15–612)

Total discontinuation, n			
AEs	5	1	4
PMF-BP	3	0	3

Patients Alive/Dead, n	3/7	2/2	1/5

Median OS, months (range)	67 (21–227)	93 (65–219)	35.5 (14–117)

**Table 2:** DSX dosing and exposure in all patient’s collection, hematologic responder (HR) and non-responder (NR) patients.

Characteristic	All patients (n=10)	HR patients (n=4)	NR patients (n=6)

Median dosage of DSX (range):			
mg/day	750 (500–1500)	812 (500–1000)	750 (500–1500)
mg/kg/day	10 mg/kg/day	12 mg/kg/day	10 mg/kg/day

Median DSX exposure, months (range)	11 (1–33)	14.5 (10–33)	6.5 (1–20)

Median number of RBC transfusion during ICT (range)	23.5 (2–300)	37.5 (2–300)	23.5 (2–76)

Median daily iron intake during ICT mg/kg/day (range)	0.47 (0.03–1.63)	0.65 (0.03–1.63)	0.47 (0.39–1.07)
